# What you see is not always what you get: the influence of epidemiology on ordering practices for herpes simplex virus and varicella zoster virus

**DOI:** 10.1128/asmcr.00112-25

**Published:** 2025-09-03

**Authors:** Blake W. Buchan, Preeti Pancholi

**Affiliations:** 1 The Medical College of Wisconsin and Children’s Wisconsin 5506, Milwaukee, Wisconsin, USA; 2 The Ohio State University Wexner Medical Center 12306, Columbus, Ohio, USA; Rush University Medical Center, Chicago, Illinois, USA

**Keywords:** ulcerative lesion, varicella zoster virus, herpes simplex virus

## Abstract

**Background:**

Herpes simplex virus (HSV) and varicella zoster virus (VZV) are common causes of clinically indistinguishable lesions. The epidemiology and clinical presentation, including anatomic site of lesion and patient age, differ between these viruses, which may influence physician ordering practices. Importantly, atypical presentation of either HSV or VZV can result in the potential for misdiagnosis or missed diagnosis.

**Case Summary:**

Two cases are presented illustrating atypical anatomic location and presentation of HSV-1 and VZV lesions that resulted in failure to consider the virus when placing orders for laboratory testing, leading to missed or misdiagnosis.

**Conclusion:**

Atypical presentation of HSV or VZV resulting in failure to order the appropriate laboratory test impacts a significant minority of cases, which can lead to failure to prescribe appropriate therapy, extended symptoms, and additional interaction with the healthcare system.

## INTRODUCTION

Herpes simplex virus (HSV) and varicella zoster virus (VZV) are human pathogens in the Alphaherpesvirinae subfamily (1, 2). Primary infection with these double-stranded DNA viruses is often associated with the development of vesicular “herpetic” lesions, followed by lifelong latency characterized by periods of asymptomatic and symptomatic viral reactivation (1, 3). All three viruses establish latency within sensory neurons; however, the clinical presentation and anatomic location of recurrent infection is influenced by both the cellular tropism of each virus and initial site of infection (1, 3). Primary or recurrent lesions caused by HSV-1 and HSV-2 typically appear in localized clusters and are visually indistinguishable (4, 5). Lesions caused by VZV appear similar to those caused by HSV though may be distinguished by their distribution across an entire dermatome, and the presence of multiple lesions at various stages of development (vesicular, ulcerative, crusted) (6). Typical characteristics of HSV and VZV are presented in Table 1. Importantly, lesion appearance and presentation may be atypical. This is particularly evident in immunocompromised patients (7, 8) and individuals with darker pigmented skin (9), which may contribute to racial disparities in diagnosis of HSV and other infections in this population (10). Further complicating clinical diagnosis is a shifting epidemiology. While historically associated with oral lesions (cold sores), HSV-1 is now a more common cause of primary genital infection than HSV-2 in some populations (11), and the impact of widespread varicella zoster vaccination has reduced the incidence of these infections in the pediatric population (12).

**TABLE 1:** Clinicopathologic features of herpes simplex and varicella zoster viruses^*a*^

Characteristic	HSV-1	HSV-2	VZV
Route of transmission	Direct contact with virus in saliva, wet lesions, or genital secretions		Direct contact or airborne transmission of respiratory secretions or wet lesions
Site of latency	Head and neck ganglia including trigeminal (cranial), olfactory, and vestibular nerve	Sacral and dorsal root ganglia	Head and neck ganglia, dorsal root sensory ganglia
Seropositivity	50-80% by adulthood	5-20% by adulthood	>90% by age 17
Primary infection	Often asymptomatic or subclinical; may develop one or a few ulcerative lesions at site of infection		Widespread rash including vesicular lesions lasting 2–3 weeks (chickenpox) accompanied by fever
Location of recurrent lesions (typical presentation)	Most commonly orolabial (cold sores), facial, and ocular; reactivation in genital location most common following primary genital infection	Localized clusters of lesions; most commonly genital (vaginal, labial, penile, rectal), reactivation in oral location uncommon	Diffuse rash typically involving a single dermatomal distribution, most commonly thoracic (T3-L2), cervical (impacting upper back), and trigeminal (zoster ophthalmicus); may rarely impact multiple dermatomes; frequently associated with severe neuropathic pain
Lesion appearance (typical presentation)	Localized clusters of lesions; typically same developmental stage (vesicular, ulcerative, crusted)		Multiple lesions at various stages of development (vesicular, ulcerative, crusted); distributed broadly across an entire dermatome
Systemic infections	Meningitis, sepsis, hepatitis, retinitis		Meningitis, visceral disease, hepatitis, pneumonitis

^*a*^ Data summarized from references 1, 3, 6, 11, 13.

The similarity in appearance of lesions, atypical presentations, and reliance on typical epidemiology can result in diagnostic error, including missed and misdiagnosis. Prior studies have reported detection of VZV in 3%–11% of genital ulcerative lesions (14–16), and a potential to miss up to 5% of both VZV and HSV infections if relying on targeted testing for HSV or VZV alone (16). Indeed, current guidelines suggest testing for HSV-1, HSV-2, and VZV from mucocutaneous swabs and generic lesion swabs in certain patient populations (17). The following cases highlight the risk of overreliance on epidemiology when selecting diagnostic tests for assessment of papular, vesicular, or ulcerative lesions and the impact to patient care resulting from missed or misdiagnosis. We provide data demonstrating age and anatomic location-based ordering bias for individual HSV and VZV nucleic acid amplification tests (NAATs) and discuss the potential value of multiplexed assays for the identification of these viruses.

## CASE PRESENTATION

### Case 1

A 43-year-old female presented with a 2–3 day history of mild dysuria without vaginal discharge and a single 3–4-mm papular lesion on her left lower labia majora. No fluctuance or drainage was noted, and no vesicular lesions or surrounding erythema were present. The patient reported the lesion had been irritable and itchy, but not painful. Her sexual partner self-reported “cold sores” and suspected HSV lesions on their face and genital region; however, laboratory testing was not pursued. The examining physician noted the lesion as appearing inconsistent with HSV; however, because of concern for exposure, a swab specimen was obtained for HSV NAAT along with serum for Rapid Plasma Reagin (RPR) to rule out chancre (primary syphilis). Given concern for a sexually transmitted infection, appropriate specimens were also obtained to test for *N. gonorrhoeae* (NG) and *C. trachomatis* (CT) infection, *Trichomonas*, and HIV. The patient was discharged with a prescription for oral cephalexin due to potential bacterial folliculitis while awaiting test results. Standard of care results were negative for HSV, RPR, CT, NG, and *Trichomonas*. Urine analysis was positive for bacteria, leukocyte esterase, and nitrite confirming a UTI; however, urine cultures were not obtained. Testing conducted using a multiplexed HSV + VZV NAAT for research purposes revealed positivity for VZV.

### Case 2

A 59-year-old male presented with a 7-day history of progressive burning and pruritic rash under both breast areas. The rash was composed of many small (1–2 mm) papular and vesicular lesions with mild erythema. Upon examination, no other rashes were present on his body. The examining physician noted a favored diagnosis of bacterial folliculitis and/or intertrigo fungal infection; however, collected a swab for VZV NAAT in addition to bacterial culture. VZV NAAT was ordered primarily to rule this out as a potential etiology given the patient age and appearance of lesions. Results from the VZV NAAT were negative, and the bacterial culture yielded moderate methicillin-resistant *S. aureus*. The patient was prescribed doxycycline for presumed bacterial folliculitis but re-presented 4 days later with persistent symptoms and expanding rash. He was prescribed over-the-counter anti-itch medication and topical steroid and was referred to dermatology. Upon presentation to the dermatology clinic 7 days later, the physician suspected a potential HSV infection, but the lesions had become crusted and were healing with noted resolution of symptoms. Testing conducted on the initial specimen using a multiplexed HSV + VZV NAAT for research purposes revealed positivity for HSV-1.

## DISCUSSION

The two cases presented typify the difficulty in discriminating HSV and VZV infections from each other as well as from common bacterial infections based on patient history and clinical presentation. These missed or misdiagnosed infections resulted in ineffective and unnecessary antibiotic therapy and contributed to persistent symptoms and discomfort that may have been shortened by appropriate antivirals. In addition, missed or misdiagnosis of HSV and VZV can have implications for reducing transmission of these pathogens through administration of antiviral prophylaxis and can impact appropriate isolation precautions for inpatients (i.e., VZV is airborne vs HSV is contact). While it is clear that these infections may be missed with current testing strategies, the frequency of missed diagnostic opportunities and the impact of patient age or anatomic site of the lesions on clinical test orders is not well characterized.

We set out to conduct a method comparison study between a multiplexed HSV + VZV NAAT (Savanna HSV 1 + 2/VZV, QuidelOrtho) and standalone HSV and VZV NAATs (Simplexa HSV 1&2 Direct and Simplexa VZV Swab Direct, Diasorin) but also collected information, including patient age, anatomic location of suspect lesions, and standard of care test orders to better understand factors impacting physician ordering practices and assess the potential impact of co-testing all suspected lesions for HSV and VZV. The study included 721 total lesion swab specimens with a clinical order for HSV or VZV NAAT across two primary adult hospitals and one primary children’s hospital located in the midwest and northeastern United States. Importantly, inclusion criteria for the assay comparison study were based on physician order with a goal to enroll at least 50 positive specimens for each target (HSV-1, HSV-2, VZV). This design provided the opportunity to examine ordering practices and lesion site in aggregate; however, because specimen enrollment was not prospective “all-comer” viral positivity data for HSV and VZV had to be considered separately.

**Fig 1:**
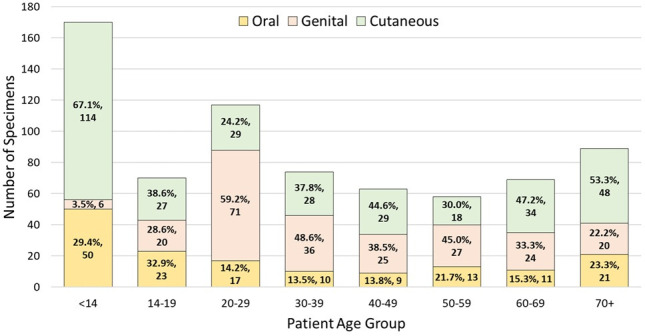
Lesion location by age group. The proportions of lesions tested from oral, genital, and cutaneous (periocular, nose/nasal, limbs, trunk, etc.) sites were similar across most decades of life, with the exception of ages 20–29, where 59.2% of specimens tested were genital sites, and ages <14, in which 67.1% of specimens tested were cutaneous sites.

The proportion of all oral, genital, and other cutaneous lesion sites (e.g., periocular, nose/nasal, limbs, trunk, etc.) tested in this study, regardless of positivity, is presented in Fig. 1. These data highlight the difference in presentation of suspected herpetic infections by age, e.g., testing for HSV and VZV is rarely considered in genital sources from patients <14 years of age while genital sources predominate in patients age 20–29 years. The distribution of lesion sources tested in all other decades of life was relatively consistent.

**Fig 2:**
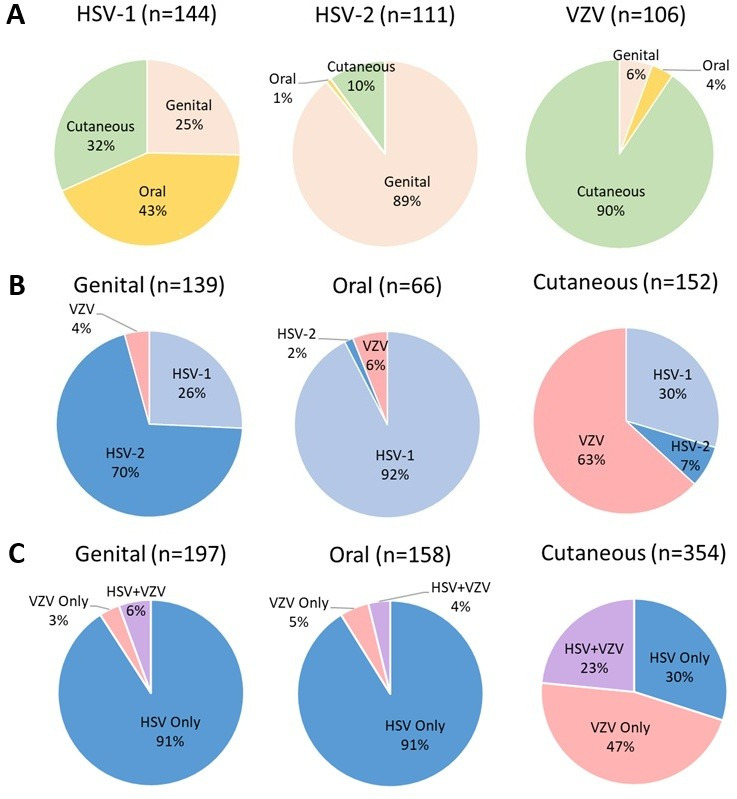
Virus positivity and physician order by lesion site. Viral positivity by anatomic site (**A**), site positivity by virus (**B**), and physician standard of care (SOC) order by anatomic site (**C**) demonstrate that test ordering largely reflects viral positivity for lesions at oral, genital, and cutaneous sites.

We next examined just those specimens with a positive result to examine epidemiology and physician ordering practice by lesion site. Among HSV-1-positive lesions, no single anatomic site reached a majority with 43% oral, 32% cutaneous, and 25% genital sites. This is contrasted with lesions positive for HSV-2, which were predominantly genital sites (89%), and lesions positive for VZV, which were predominantly cutaneous sites (90%) (Fig. 2A). We next examined lesion site positivity by virus. Among all positive lesions, oral sites were predominantly positive for HSV-1 (92%), with only 2% of positive oral lesions attributed to HSV-2 and 6% VZV. In contrast, 70% of positive genital lesions were attributed to HSV-2, 26% HSV-1, and just 4% VZV. The distribution of viruses among positive cutaneous lesions was more evenly spread with 63% VZV, 30% HSV-1, and 7% HSV-2 (Fig. 2B). Physician ordering practices were largely reflective of this source-based epidemiology. Among genital lesions, 90.1% of test orders were for HSV alone, 5.6% of orders for HSV and VZV, and just 3.6% of orders for VZV alone (Fig. 2C). Similarly, for oral lesions, 91.1% of orders were for HSV alone, with 3.8% for HSV and VZV and 5.1% for VZV alone. Test orders for cutaneous lesions more frequently included VZV, with 46.6% of orders for VZV alone, 23.4% for VZV and HSV, and 29.9% for both HSV alone (Fig. 2C).

**Fig 3:**
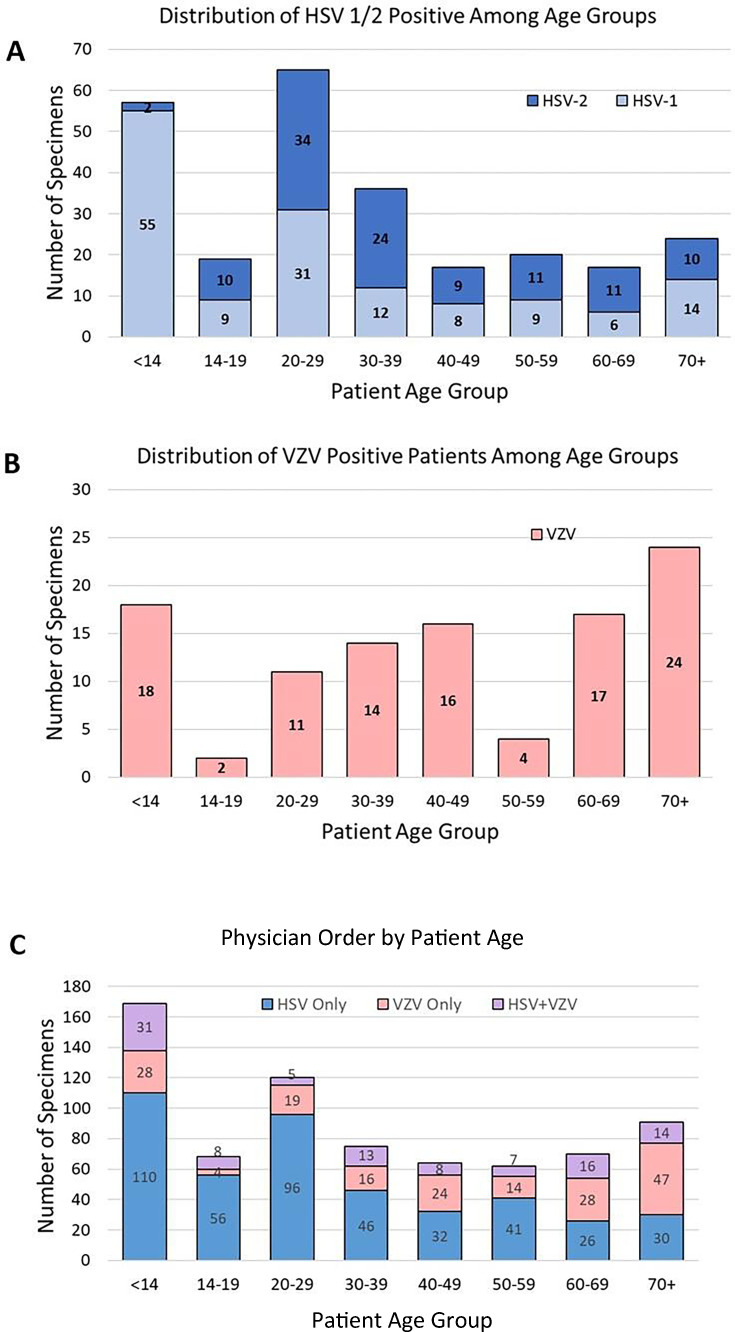
Virus positivity and physician order by age. Virus positivity by anatomic age for HSV (**A**) and VZV (**B**) compared to physician standard of care (SOC) order (**C**) demonstrates that test ordering largely reflects virus positivity by age group.

Finally, we examined the specimens with a positive result to assess the correlation of age-based epidemiology and physician ordering practices. HSV-positive lesions were most prevalent in patients <14 years of age (56/254, 22.0%), where 96.4% were HSV-1, and in patients aged 20–29 years (65/254, 25.5%), where HSV-1 and HSV-2 equally represented (Fig. 3A). VZV positivity was also frequent in patients <14 years of age (17/105, 16.2%), dropped dramatically in those 14–19 years, and then gradually increased with age, reaching a peak in those age >70 (24/105, 22.8%) (Fig. 3B). Physician ordering practices were somewhat reflective of this age-based epidemiology, with 34.9% (59/169) of orders, including VZV either alone or in conjunction with HSV for individuals <14 years of age and 67.0% (61/91) for individuals <70 years of age. Conversely, VZV was only ordered for 17.6% (12/68) patients aged 14–19 years and 20% (24/120) of patients aged 20–29 years, reflecting the lower prevalence of VZV-positive lesions in these age groups (Fig. 3C).

Importantly, though physician ordering was generally aligned with anatomic site-based and age-based epidemiology, there were instances of missed diagnosis resulting from failure to order a test for the virus ultimately found to be causing the lesion. During our study, we identified 3.1% (11/358) patients for which there was not a physician order placed for the virus that was found to be present in the lesion. This was most commonly observed in cases of cutaneous lesions in patients >50 years of age for which only VZV PCR was ordered, but the lesion was found to be positive for HSV (Table 2). The impact of these missed diagnoses included misdiagnosis as a bacterial infection with prescription of unnecessary antibiotics and missed diagnosis with failure to prescribe antivirals, which in some cases resulted in persistent symptoms and additional interaction with the healthcare system. In other cases, empiric antiviral treatment was prescribed that resolved symptoms, though therapy was based on incorrect diagnosis. Of note, only 14.3% (103/721) of all cases included physician orders for both HSV and VZV tests. Our findings confirm previous reports demonstrating a small but significant minority of HSV- and VZV-positive lesions with atypical presentation (e.g., VZV-positive genital sources, HSV-positive cutaneous sites) (14–16) and provide further insight to epidemiologic factors that may drive physician ordering practices.

**TABLE 2:** Cases of missed diagnosis resulting from targeted standard of care order

Age (yr)/sex^*c*^	Lesion site	SOC order	Virus detected	Synopsis and impact
SOC order for HSV, testing detected VZV				
43/f	Genital	HSV	VZV	Presented with dysuria and popular lesion on labia majora; sexual partner with history of facial lesions presumed to be HSV; order for HSV PCR and other sexually transmitted infections based on history, prescribed cephalexin for presumed bacterial folliculitis^*a*^
17/m	Cutaneous	HSV	VZV	Presented with vesicular rash on left upper arm and finger; prescribed acyclovir empirically for presumed HSV infection; no further follow-up after negative SOC HSV result
71/f	Oral	HSV	VZV	Presented with scabbing lesions on lips; no other clinical or social history provided
SOC order for VZV, testing detected HSV				
59/m	Cutaneous	VZV	HSV-1	Presented with burning pruritic rash on chest; bacterial culture ordered at same time as VZV PCR grew *Staphylococcus aureus*; patient was prescribed doxycycline for presumed bacterial infection; after failing to respond to antibiotics, patient was referred to dermatology, but no further testing was pursued^*b*^
55/f	Cutaneous	VZV	HSV-2	Presented with history of type 2 diabetes and multiple vesicular lesions on face and scalp; bacterial culture ordered at same time as VZV PCR grew MRSA; patient was prescribed Bactrim for presumed bacterial infection
73/m	Cutaneous	VZV	HSV-1	Presented with grouped vesicles with erythematous base on right lateral neck; patient also had a hemorrhagic crusted ulcer on left lower lip; patient was started on empiric Valtrex
58/m	Cutaneous	VZV	HSV-1	Presented with diffuse painful papular rash that initially started in the groin and quickly progressed to involve legs, arms, fingers, chest/trunk, face, and back; patient was started on IV vancomycin and acyclovir
72/m	Cutaneous	VZV	HSV-1	Presented with one week of painful vesicular rash on the face including serous and hemorrhagic crusted vesicular papules and plaques on the forehead, cheeks, nose, and chin involvement; upon exam, left ocular erythema and tearing were noted; physician note indicated a primary differential of disseminated zoster with ophthalmologic involvement; patient received IV acyclovir with no follow-up testing
56/f	Cutaneous	VZV	HSV-2	Presented with two satellite vesicles and a scalloped erosion over the central sacrum and was referred to Dermatology; exam noted several angulated erosions along superior gluteal cleft with contralateral satellite lesion and a few vesicles at superior border; Valtrex therapy was initiated
58/f	Cutaneous	VZV	HSV-2	Presented with grouped vesicles with an erythematous base on right lower back; clinical appearance, location, and distribution noted to be consistent with zoster; patient had prior shingles infection and had received the Shingrix vaccine a few weeks prior; patient was prescribed Valtrex for presumed vaccine or natural VZV reactivation
43/f	Cutaneous	VZV	VZV and HSV-2	Presented with multiple lesions on scalp and forehead; standard of care (SOC) test order for VZV PCR was positive; patient was prescribed Valtrex for presumed VZV infection

^*a*^ Presented as Case 1 in this article.

^*b*^ Presented as Case 2 in this article.

^*c*^ m, male; f, female.

There are currently four FDA-cleared tests that incorporate multiplex detection and identification of HSV-1, HSV-2, and VZV in a single assay. The Lyra HSV 1 + 2/VZV (QuidelOrtho) is a high complexity assay best suited for larger batch-based testing and is compatible with several real-time thermocyclers (15). The Alinity m HSV 1 + 2/VZV assay is a moderate complexity assay performed on the high throughput Alinity m analyzer. The Savanna HSV 1 + 2/VZV assay (QuidelOrtho) is a moderate complexity, on-demand cassette-based assay performed on the sample-to-answer Savanna analyzer (18). Implementation of a multiplexed HSV + VZV assay would ensure simultaneous testing for HSV and VZV in lesion swabs, thereby reducing the reliance on clinical and epidemiologic criteria to inform ordering and increasing laboratory diagnosis by up to 3.4% based on our data. This is similar to the approach taken by other multiplexed assays intended to identify multiple pathogens associated with a similar clinical presentation, such as influenza, RSV, and SARS-CoV-2 in respiratory specimens or *N. gonorrhoeae* and *C. trachomatis* in urogenital specimens.

The benefits of a multiplexed HSV + VZV testing approach must be balanced against potential disadvantages to ensure a net value for the patient and healthcare system. The cost of care, inclusive of higher test cost and poor reimbursement, is a commonly cited barrier to multiplexed syndromic testing. Currently, no multiplex reimbursement codes exist for HSV and VZV combination assays, so each agent is charged individually. Therefore, patients with a low risk of HSV or VZV would potentially incur unnecessary cost if only combination testing was available. Both the Savanna HSV 1 + 2/VZV assay and Alinity m HSV 1 + 2/VZV assay provide the option for selective reporting to accommodate individual HSV and VZV orders as well as combinational testing. Counterbalancing the increased initial cost of combined HSV + VZV testing is the cost of unnecessary antibiotics, additional visits to healthcare facilities, and lost productivity due to missed or misdiagnosed infections. Further, the differentiation of HSV-1 from HSV-2 can have prognostic implications for the expected frequency of recurrence and risk of transmission, i.e., recurrence of HSV-2 at genital sites and HSV-1 at oral sites far exceeds that of HSV-2 at oral sites or HSV-1 at genital sites (19).

In conclusion, the cases and data presented herein demonstrate the challenges in the diagnosis of lesions resulting from HSV and VZV infection. Ultimately, each healthcare system and laboratory must weigh financial, population health, and patient care considerations when selecting a diagnostic approach for evaluation of patients with potential HSV or VZV infections.
